# Classification and Recognition of Soybean Quality Based on Hyperspectral Imaging and Random Forest Methods

**DOI:** 10.3390/s25051539

**Published:** 2025-03-01

**Authors:** Man Chen, Zhichang Chang, Chengqian Jin, Gong Cheng, Shiguo Wang, Youliang Ni

**Affiliations:** 1 Nanjing Institute of Agricultural Mechanization, Ministry of Agriculture and Rural Affairs, Nanjing 210014, China; chenman@caas.cn (M.C.); charlie728@163.com (Z.C.);; 2 National Digital Agriculture Equipment (South China Intelligence Agricultural Mchine) Innovation Sub-Center, Nanjing 210014, China; 3 Xinjiang Academy of Agricultural Reclamation Sciences, Institute of Mechanical Equipment, Shihezi 832000, China; wansg@163.com

**Keywords:** soybean, hyperspectral imaging, feature extraction, random forest, classification and identification

## Abstract

To achieve the rapid and accurate classification and identification of soybean components, this study selected soybeans harvested by the 4LZ-1.5 soybean combine harvester as the research subject. Hyperspectral images of soybean samples were collected using the Pika L spectrometer, and spectral information was extracted from the regions of interest (ROI) in the images. Eight preprocessing methods, including baseline correction (BC), moving average (MA), Savitzky–Golay derivative (SGD), normalization, standard normal variate transformation (SNV), multiplicative scatter correction (MSC), first derivative (DS), and Savitzky–Golay smoothing (SGS), were applied to the raw spectral data to eliminate irrelevant information. Feature wavelengths were selected using the successive projections algorithm (SPA) and the competitive adaptive reweighted sampling (CARS) algorithm to reduce spectral redundancy and enhance model detection performance, retaining eight and ten feature wavelengths, respectively. Subsequently, a random forest (RF) model was developed for soybean component classification. The model parameters were optimized using particle swarm optimization (PSO) and differential evolution (DE) algorithms to improve performance. Experimental results showed that the RF classification model based on SPA-BC preprocessed spectra and DE-tuned parameters achieved an optimal prediction accuracy of 1.0000 during training. This study demonstrates the feasibility of using hyperspectral imaging technology for the rapid and accurate detection of soybean components, providing technical support for the assessment of breakage and impurity levels during soybean harvesting and storage processes. It also offers a reference for the development of future machine-harvested soybean breakage and impurity detection systems.

## 1. Introduction

Soybeans are a globally important grain and economic crop [1]. The mechanization and intelligentization of the entire soybean industry chain, including planting, harvesting, and processing, have significantly advanced agricultural modernization [2]. During soybean harvesting, the real-time detection of the breakage rate and impurity rate is crucial, as these factors directly affect the quality of the harvesting process, economic benefits, and the efficiency of subsequent processing stages. High breakage rates reduce the market value of soybeans, especially when used for consumption or as seeds, where integrity is critical [3]. Real-time detection enables the timely adjustment of harvesting parameters to minimize breakage rates. Similarly, high impurity rates increase the cost of post-harvest cleaning and processing. Real-time detection can optimize the performance of cleaning devices in harvesters, improve separation efficiency, and reduce impurity rates. At present, manual sampling is commonly used for soybean crushing and impurity detection, with inherent problems such as high labor intensity and large errors. Manual sampling and testing can only achieve a quantitative evaluation of harvesting quality, but cannot achieve the accurate measurement of harvesting quality in large quantities and in real time. It cannot meet the real-time detection requirements for crushing and impurities in soybean mechanized harvesting.

Currently, the online detection of the soybean breakage rate and the impurity rate primarily relies on technologies such as vision, spectroscopy, and lasers [4,5,6,7,8]. Machine-vision-based detection involves capturing soybean sample images using high-speed or industrial cameras, applying image processing algorithms, and leveraging features like color, shape, and texture to distinguish intact soybeans, broken particles, and impurities. Md Abdul Momin et al. developed a soybean quality detection model based on machine vision. The algorithms successfully identified the dockage fractions, with an accuracy of 96% for split beans, 75% for contaminated beans, and 98% for both defect beans and stem/pods [9]. Li bo et al. utilized hyperspectral imaging technology to identify four varieties of soybean seeds. The high-level data fusion, based on Bayesian consensus, provided the optimal results, with an accuracy and F1-score of 93.13% and 93.70% in the prediction phase, respectively [10]. Lin Wei et al. proposed a deep-learning-based method for the online classification of soybean seeds. The F-score of the normal, damaged, abnormal, and non-classifiable soybeans reached about 95.97%, 97.41%, 97.25%, and 96.14%, respectively [11]. Song Chenxu et al. proposed an algorithm for extracting multiple phenotypic parameters of soybean grains, allowing for the simultaneous acquisition of various phenotypic traits from soybean images and quality assessment. The correct recognition rate for soybean grains was 95.2%, while the recognition rates for damaged and diseased soybeans were 91.25% and 88.94%, respectively [12]. However, machine vision detection requires stable lighting conditions and uniform sample distribution, and additional optimization is needed to address occlusion issues in complex scenarios.

Spectroscopic analysis, leveraging differences in characteristic absorption spectra in the near-infrared, mid-infrared, or hyperspectral range, is capable of detecting soybean composition and the proportion of broken particles, with strong discrimination for various impurities and breakage rates. Chen Man et al. applied inter-band autocorrelation analysis to identify the feature wavelengths of soybean spectra for different indicators. They used support vector regression to build an inversion model for impurity rates, achieving modeling determination coefficients >0.86, validation determination coefficients >0.79, root mean square errors <0.32, and relative analysis errors >1.7 [13]. Luo Wang et al. proposed a soybean recognition method based on temporal spectral standard curves, achieving an overall classification accuracy of 86.95%, user accuracy of 90.91%, mapping accuracy of 86.14%, and an F1-score of 0.8846 [14]. Li Wei et al. established a rapid identification model for soybean quality using Raman spectroscopy, combined with a characteristic wavelength extraction method, with an accuracy rate of 100% [15]. However, research on classifying and identifying soybean components using spectral imaging systems for machine-harvested soybean sample images remains insufficiently explored. Prominent in: the specific spectral bands that can effectively differentiate the compositional variations in soybeans remain to be clearly identified. Effective data preprocessing methods can achieve data dimensionality reduction while ensuring detection accuracy. This study will address these issues.

This study focuses on soybean samples harvested by the 4LZ-1.5 soybean combine harvester. Through indoor physicochemical analysis and spectral measurement, spectral information from the regions of interest in the hyperspectral images was extracted and preprocessed. A random forest classification model for soybean component identification was then established. Dimensionality reduction was performed using the successive projections algorithm and competitive adaptive reweighted sampling to select the feature wavelengths. Based on these feature wavelengths, an RF-based soybean component classification model was developed. Furthermore, particle swarm optimization and differential evolution algorithms were introduced to optimize the hyperparameters of the RF model, enhancing its performance. This study provides a novel approach for detecting impurity and breakage content during soybean harvesting, storage, and processing.

## 2. Materials and Methods

### 2.1. Samples and Component Classification

The experimental samples were sourced from the soybean planting base in Houmajialing Village, Fengle Town, Zhongxiang City, Hubei Province. The experiment used the 4LZD-1.6 soybean combine harvester to harvest the variety Zhongdou 55 [16]. During the harvesting process, a total of 100 test samples were collected from the grain discharge port of the harvester. The average one hundred grain weight of the samples was 21.27 g. The moisture content ranged from 18.5% to 21.1%, with an average of 19.2%. The impurity rate ranged from 1.32% to 2.23%, with an average of 1.53%, while the breakage rate ranged from 1.48% to 3.19%, with an average of 2.27%. After collection, the samples were sealed in bags, numbered, and transported back to the laboratory for further analysis.

**Figure 1:**
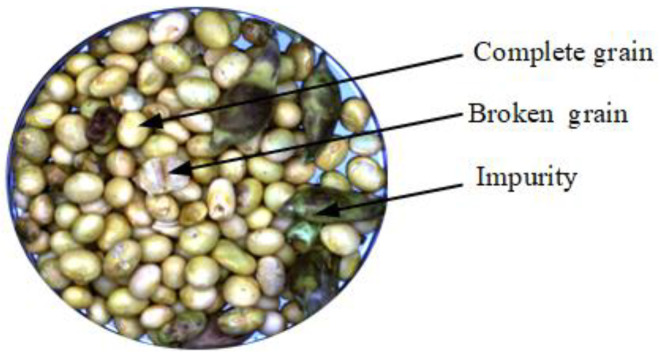
Mechanically harvested soybean components.

Due to factors such as harvesting conditions and soybean growth, mechanical harvesting of soybeans often results in breakage and contamination by impurities. In this study, soybean components are classified into three categories: intact grains, broken grains, and impurities (Figure 1). Intact grains refer to soybeans that have not suffered mechanical damage; broken grains refer to soybeans with damaged skin, cracks, or crushed seeds; and impurities refer to non-cereal materials (such as straw, soybean pods, stones, soil, etc.). In this study, the focus for the impurities is on soybean biomass contaminants, particularly straw and soybean pods.

### 2.2. Spectral Image Information Collection

**Figure 2:**
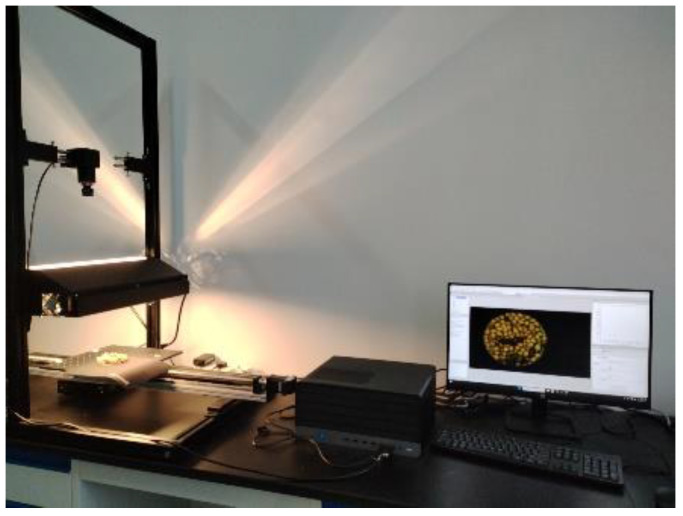
Spectral imaging system.

The spectral imaging system consists of a Pika L spectrometer (Resonon, Bozeman, MT, USA), a halogen light source, a sample stage, a sample scanning motion control system, and a computer (Figure 2). The Pika L spectral acquisition range is 400 nm to 1000 nm, with a spectral resolution of 2.7 nm, 281 spectral channels, and a sampling interval of 1.07 nm. The spectral acquisition software used is Spectronon Pro (Resonon, Bozeman, MT, USA, version 3.4.10). The sample stage moves at a speed of 3.426 cm·s^−1^ to ensure that image distortion does not occur during the line scanning process. After preheating the halogen light source for 20 min, spectral data are collected, according to the standard procedure of the spectral acquisition system. A whiteboard is used to calibrate spectral data. The dark current is corrected through preprocessing using Spectronon Pro. Three sets of images were collected for each sample by rotating it to change its relative position to the camera. A total of 300 raw spectral data were obtained.

### 2.3. Feature Spectrum Extraction

**Figure 3:**
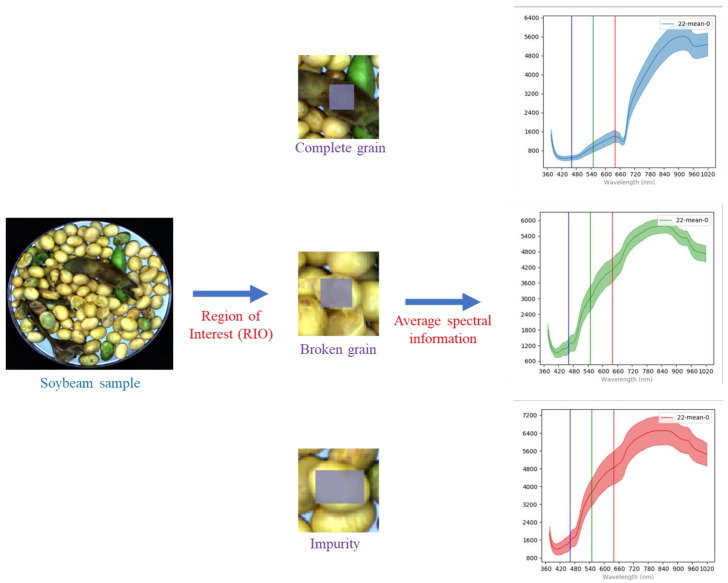
Extraction of spectral information of soybean components.

**Figure 4:**
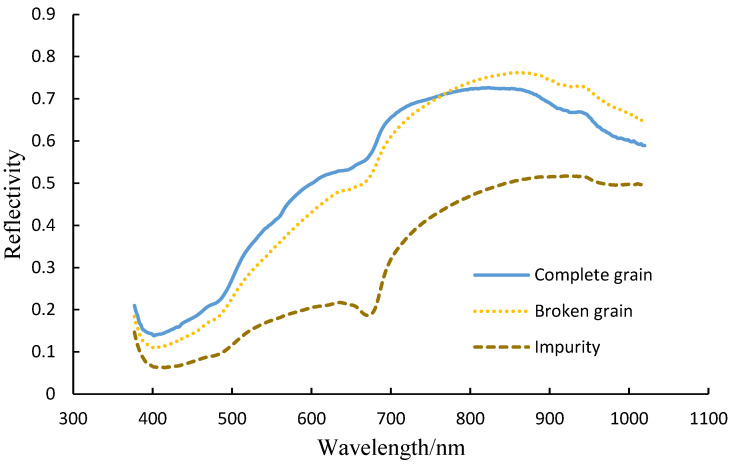
Spectral information of different components.

SpectrononPro spectral analysis software was used to extract the regions of interest (ROI) from the spectral images of intact grains, broken grains, and impurities of soybeans. As shown in Figure 3, a square region with a side length of 200 pixels was selected as the ROI, and the average spectrum of the ROI was extracted as the spectral information for that category of soybean sample. A total of 1192 sets of spectral data were extracted, including 513 sets for intact grains, 256 sets for broken grains, and 423 sets for impurities. Figure 4 shows a set of spectral information graphs for different soybean components. The surface of intact soybeans is relatively smooth and exhibits more uniform optical properties. The edges and surface structures of broken soybeans are more complex, causing more light scattering and resulting in a reflection spectrum different from that of intact soybeans. Impurities have more irregular concave and convex shapes on their surfaces, causing additional scattering of light upon contact, leading to significant changes in the spectral reflectance [17]. Furthermore, inconsistencies in the moisture content levels of the three components also contribute to differences in spectral reflectance. These variations provide valuable data support for the identification of soybean components.

### 2.4. Sample Data Preprocessing Methods

Due to the low signal-to-noise ratio (SNR) and large fluctuations in the spectral data in the 377–410 nm and 980–1019 nm bands, which leads to poor stability, data in these two wavelength ranges were removed. After eliminating the low SNR bands, the soybean sample spectral data displayed 265 bands. The original spectral data were preprocessed using eight algorithms: baseline correction, moving average, Savitzky–Golay convolution derivative, normalization, standard normal variate, multiplicative scatter correction, derivative spectroscopy, and Savitzky–Golay convolution smoothing. These methods were applied to further remove irrelevant information [18].

### 2.5. Soybean Component Classification Method Based on Spectral Information

The soybean samples were divided into calibration and prediction sets in a 4:1 ratio using the sample set partitioning based on the joint x-y distance (SPXY) method [19].

The original spectral data contains a large amount of redundant and irrelevant spectral information, as well as various noise, which reduces computational speed and affects the accuracy and precision of the model predictions. To address this, dimensionality reduction was performed using the successive projections algorithm (SPA) and competitive adaptive reweighted sampling (CARS) to select the feature wavelengths [20].

The random forest classification method was employed to build the spectral information-based soybean component classification prediction model. RF is an ensemble learning method that improves the accuracy and robustness of the overall model by constructing a large number of decision trees and combining their prediction results. In this study, the particle swarm optimization (PSO) and differential evolution (DE) algorithms were introduced to optimize the hyperparameters of the RF model, thereby enhancing the model’s performance [21]. At the same time, we established k-nearest neighbor (KNN) and support vector machine (SVM) soybean component recognition models to analyze the effectiveness of different modeling methods in soybean component recognition.

### 2.6. Accuracy Validation

In this study, the precision rate (*R*_Precision_), the recall rate (*R*_Recall_), and the comprehensive evaluation index (F1-score, *F*_1_) [22] were used as evaluation indicators of the model’s performance,

(1) $$R_{Precision}=\frac{T_{P}}{\left(T_{P}+F_{P}\right)},$$

(2) $$R_{Recall}=\frac{T_{P}}{\left(T_{P}+F_{N}\right)},$$

(3) $$F_{1}=\frac{2\times R_{Precision}\times R_{Recall}}{R_{Precision}+R_{Recall}},$$

where *R*_Precision_ represents the precision rate; *R*_Recall_ represents the recall rate; *F*_1_ represents the comprehensive evaluation index F1-score; *T*_P_ represents the number of correctly classified soybean samples predicted; *F*_P_ represents the wrongly classified soybean samples predicted; *F*_N_ represents the correctly classified soybean samples predicted to be misclassified soybean samples.

## 3. Results and Discussion

### 3.1. Spectral Data Preprocessing

In this experiment, spectral data from 1192 soybean sample images were extracted. Based on this spectral dataset, RF soybean component classification models were established using the original spectra and eight types of preprocessed spectra. The results are shown in Table 1.

From Table 1, it can be seen that the RF soybean component classification model based on the original near-infrared spectra achieved the following performance metrics: for intact grains, the precision, recall, and F1-score were 0.9867, 0.9801, and 0.9843, respectively; for broken grains, the precision, recall, and F1-score were 0.9375, 0.9574, and 0.9474, respectively; and for impurities, the precision, recall, and F1-score were all 1.0, resulting in an overall classification accuracy of 0.9790. This indicates that the model exhibits stable performance in regards to soybean component classification.

After preprocessing with methods such as baseline correction, Savitzky–Golay convolution derivative, normalization, and derivative spectroscopy, the overall classification accuracy of the model was higher than that of the model based on the original spectra, with all accuracy rates above 0.9900. Among the preprocessed spectra, the two methods (BC and normalization) with the best performance were selected for further model processing. The different classification results are related to the different data processing principles of each preprocessing method. Baseline calibration effectively removes baseline offset or drift caused by instruments, sample containers, or other factors, ensuring that the collected spectra reflect the characteristics of the soybean samples themselves and improving data reliability. Filtering techniques such as moving average and Savitzky–Golay filtering are used to reduce random noise in the spectral data and suppress the impact of noise on the detection results. Normalization adjusts the intensity ratio of the spectra to the same level, which facilitates comparisons between data. Standard normal variable transformation and multivariate scattering correction can correct scattering effects and reduce the impact of scattering on the detection results. Derivative spectroscopy highlights the details of teh spectral curves by calculating first-order, second-order, or higher-order derivatives, which helps to separate overlapping peaks and eliminate the influence of baseline drift. Deconvolution is used to separate overlapping signals in the spectra to obtain clearer peak information.

Because the spectral data of impurities in the samples differ significantly from those of the soybean grains, the RF model was able to achieve accurate identification for all different preprocessing methods. During the mechanized harvesting process, intact grains come into direct contact with harvesting machine components, and mechanical damage leads to surface damage and crushing, turning them into broken grains. However, there is no fundamental difference in the composition between intact and broken grains. Therefore, the spectral data of intact and broken grains overlap, which is the main cause of misclassification between these two categories.

**Figure 5:**
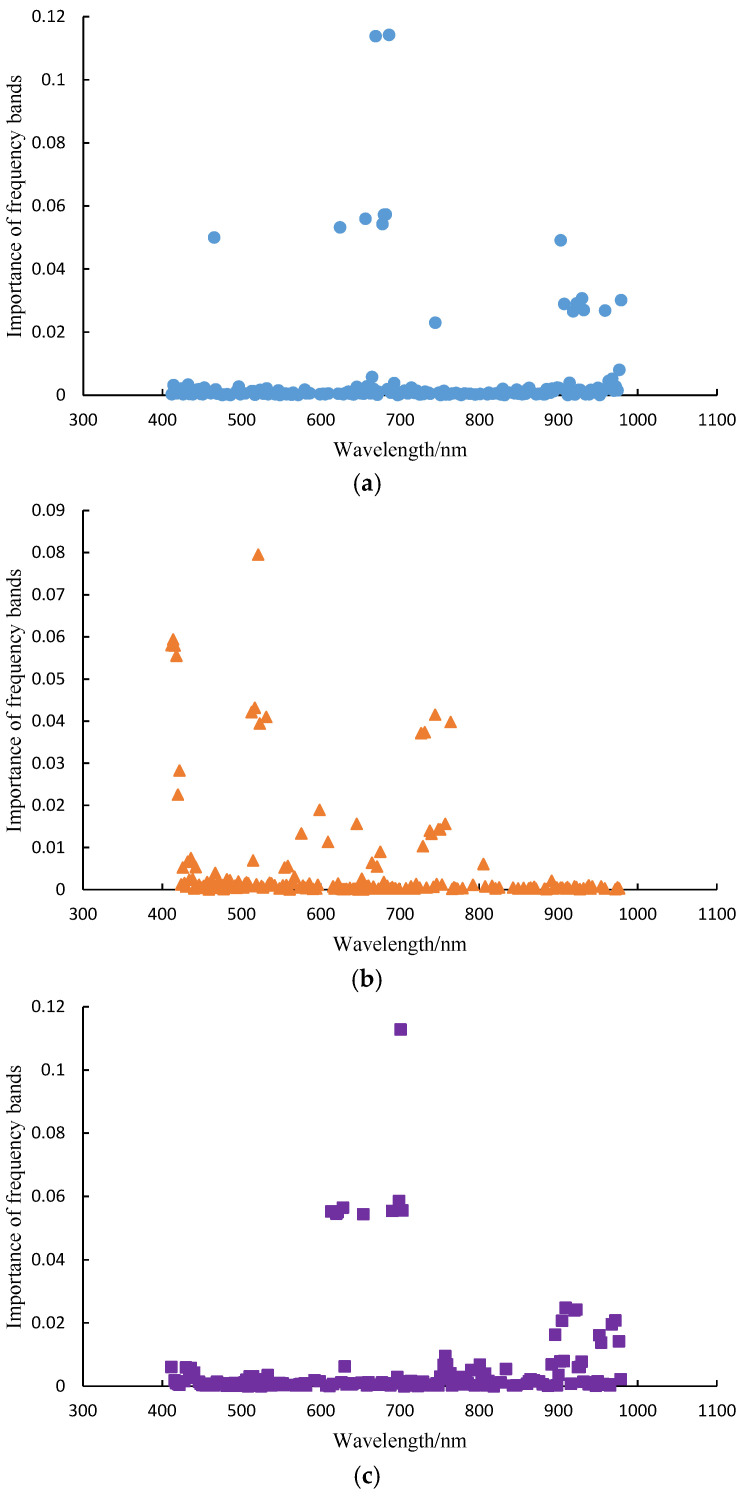
The importance of different preprocessed spectral modeling bands. (**a**) Raw data; (**b**) BC; (**c**) normalization.

Figure 5 shows the spectral band importance when constructing the RF soybean component classification model, based on different preprocessed spectral data. In the modeling process using the original data, the top three spectral bands with the highest contribution rates were 686.12 nm, 668.96 nm, and 681.84 nm, with contribution rates of 0.1143, 0.1138, and 0.0573, respectively. The cumulative contribution rate was 0.2854. For the BC preprocessed spectral data, the top three spectral bands with the highest contribution rates were 521.08 nm, 413.81 nm, and 411.77 nm, with contribution rates of 0.0795, 0.0594, and 0.0581, respectively. The cumulative contribution rate was 0.1970. For the normalization preprocessed spectral data, the top three spectral bands with the highest contribution rates were 701.18 nm, 699.03 nm, and 628.43 nm, with contribution rates of 0.1128, 0.0586, and 0.0565, respectively. The cumulative contribution rate was 0.2279. From this, it can be seen that among all the spectral bands, some bands play a decisive role in modeling, while others have no positive significance for modeling. Therefore, while ensuring detection accuracy, it is possible to attempt to reduce the dimensionality of the spectral data and decrease the computational complexity of the model by extracting feature wavelengths.

### 3.2. Feature Wavelength Extraction

#### 3.2.1. Successive Projections Algorithm

**Figure 6:**
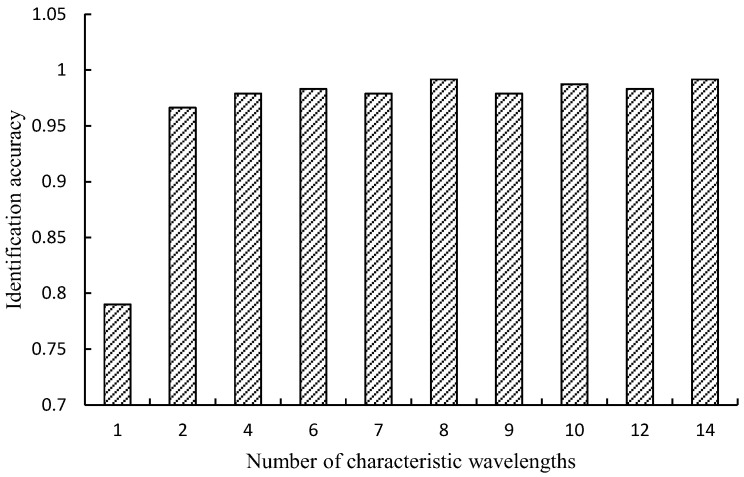
Recognition accuracy of models with different characteristic wavelength numbers (SPA).

Figure 6 illustrates the process of feature wavelength extraction using the SPA algorithm on the original spectral data. The selection process is determined by the classification accuracy of the RF soybean component classification model constructed with the selected feature wavelengths. The higher the accuracy, the better the model’s performance. As the number of feature wavelengths changes, the soybean component recognition accuracy gradually fluctuates. When the number of feature wavelengths is eight, the RF soybean component classification model achieves the highest accuracy of 0.9916.

**Figure 7:**
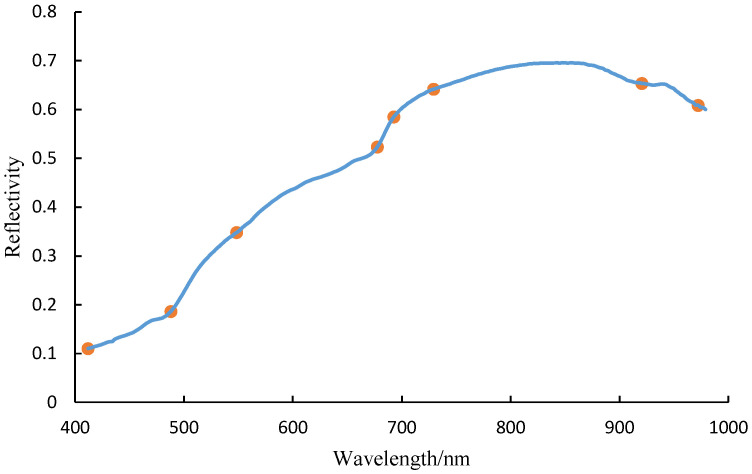
The optimal feature wavelength obtained through SPA.

Using the SPA algorithm to extract feature wavelengths from the original spectral data, the optimal feature wavelengths obtained are shown in Figure 7. The eight feature wavelengths are 677.54 nm, 920.64 nm, 729.25 nm, 487.84 nm, 548.24 nm, 972.27 nm, 692.58 nm, and 411.77 nm, accounting for 3.02% of the total wavelength range.

#### 3.2.2. Competitive Adaptive Reweighted Sampling Algorithm

**Figure 8:**
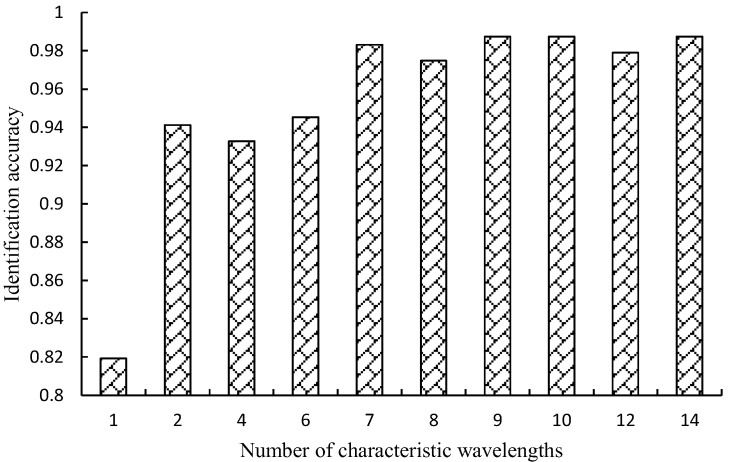
Recognition accuracy of models with different characteristic wavelength numbers (CARS).

**Figure 9:**
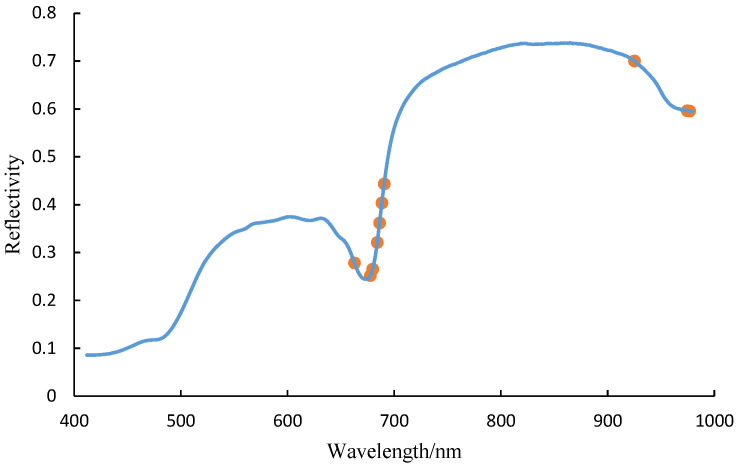
The optimal feature wavelength obtained through CARS.

Figure 8 shows the process of extracting feature wavelengths from the original spectral data using the CARS algorithm. When the number of feature wavelengths is 10, the RF soybean composition classification model achieves the highest accuracy of 0.9874. The optimal feature wavelengths obtained using the CARS algorithm are shown in Figure 9. The 10 feature wavelengths are 688.28 nm, 683.98 nm, 662.54 nm, 925.11 nm, 690.42 nm, 686.12 nm, 976.78 nm, 974.53 nm, 679.68 nm, and 677.54 nm, accounting for 3.77% of the total wavelength range.

#### 3.2.3. Component Recognition Based on Feature Wavelengths

The RF soybean composition classification models were established based on the characteristic wavelengths of both the original and preprocessed spectra, as shown in Table 2. The RF model, based on BC-preprocessed spectra combined with SPA, achieved the best prediction performance in the classification of the prediction set. At this point, the precision, recall, and F1-score for identifying intact soybean grains were 1.0000, 0.9899, and 0.9979, respectively. The precision, recall, and F1-score for identifying broken grains were 0.9714, 1.0000, and 0.9855, respectively. For impurities, the precision, recall, and F1-score were all 1.0000, and the overall classification accuracy was 0.9958.

### 3.3. Hyperparameter Optimization

Random forest is an ensemble learning method, based on decision trees, which improves prediction performance by constructing multiple decision trees and combining their prediction results. The performance of the model can be optimized by adjusting key parameters in the random forest, such as the number of trees, tree depth, and other factors. Therefore, this study uses the PSO and DE algorithms to fine-tune the hyperparameters of the RF model and constructs the RF soybean component classification model using the optimized hyperparameters. After hyperparameter tuning, the prediction results of each model are shown in Table 3.

The RF soybean component classification model based on SPA-BC preprocessed spectra with PSO tuning achieved an optimal training prediction accuracy of 1.00. The RF soybean component classification model based on CARS preprocessed spectra with PSO tuning achieved an optimal training prediction accuracy of 0.9916. The RF model based on SPA-BC preprocessed spectra with DE tuning also achieved an optimal training prediction accuracy of 1.00. Similarly, the RF model based on CARS preprocessed spectra with DE tuning achieved an optimal training prediction accuracy of 0.9916. By tuning the hyperparameters of the RF model using PSO and DE, the model’s classification performance on the training set was improved. The RF soybean component classification model based on SPA-BC preprocessed spectra with DE tuning achieved the best prediction performance on the test set, with the recognition accuracy for complete grains, broken grains, and impurities all reaching 1.0000.

### 3.4. Feature Wavelength Importance

**Figure 10:**
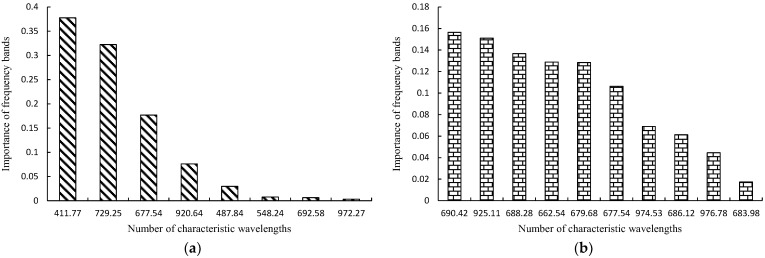
The contributions of characteristic wavelength modeling. (**a**) DE-SPA-BC; (**b**) DE-CARS.

Figure 10 shows the importance of spectral bands in the RF soybean component classification model based on DE-SPA-BC and DE-CARS preprocessed spectral data. For the DE-SPA-BC preprocessed spectral data modeling, the top three bands with the highest contribution rates were 411.77 nm, 729.25 nm, and 677.54 nm, with contribution rates of 0.3777, 0.3223, and 0.1768, respectively, resulting in a cumulative contribution rate of 0.8768. For the DE-CARS preprocessed spectral data modeling, the top three bands with the highest contribution rates were 690.42 nm, 925.11 nm, and 688.28 nm, with contribution rates of 0.1565, 0.1510, and 0.1367, respectively, resulting in a cumulative contribution rate of 0.4441.

Table 4 presents the classification results of the RF soybean component classification model constructed using the most important feature wavelengths. The overall classification accuracy of the RF soybean component classification model, constructed using the top three important bands from DE-CARS, is 0.9664. In contrast, the overall classification accuracy of the RF soybean component classification model, constructed using the top three important bands from DE-SPA-BC, is 1.0000. This indicates that although there is a slight reduction in classification accuracy when constructing the RF model based on important feature wavelengths, the accuracy remains above 0.95. This approach effectively reduces the data processing load, while maintaining a high level of classification accuracy.

### 3.5. Recognition Effectiveness of Different Models

Table 5 presents the recognition results of the soybean component classification model based on KNN and SVM. The experimental results in Table 4 and Table 5 indicate that the three recognition models have achieved consistent performance regarding impurity classification and recognition, all of which can accurately identify impurities in this batch of samples, with an F1-score reaching 1.0000. From the perspective of complete grain classification performance, the RF model performs the best, with an F1-score of 1.0000, while the KNN model has an F1-score of 0.9936, and the SVM model has an F1-score of 0.9968. From the perspective of identifying broken grains, the RF model performs the best, with an F1-score of 1.0000, while the KNN model has an F1-score of 0.9818, and the SVM model has an F1-score of 0.9908. The overall classification accuracy of the RF, KNN, and SVM models is 1.0000, 0.9916, and 0.9958, respectively. KNN identifies the k nearest neighbors to the sample in the training set and classifies them based on their categories. SVM achieves classification by constructing hyperplanes to maximize the spacing between different categories in the dataset. RF improves classification accuracy by generating multiple decision trees and combining their prediction results. The differences in classification principles among the three models result in different classification outcomes.

## 4. Discussion

Spectral technology has been widely applied in agricultural classification and identification. Liu Shuang et al. used hyperspectral imaging for soybean disease classification, employing two classifiers, the least squares support vector machine (LSSVM) and support vector machine (SVM), to establish disease classification models, achieving classification accuracies of 100% and 98.85%, respectively [23]. Zhang Hang et al. explored the feasibility of applying hyperspectral imaging technology in wheat-seed classification and identification. They established a principal component analysis–support vector machine (PCA–SVM) classification model, achieving a classification accuracy of approximately 80% [24]. Xu Da et al. investigated the spectral feature differences among four typical land cover types: soybean, maize, rice, and bare soil. They extracted diagnostic spectral features hidden in the hyperspectral data structure to achieve land cover classification and identification [25]. This study applies spectral technology to the classification and identification of soybean sample components, achieving accurate identification of intact grains, broken grains, and impurities in soybean samples.

In addition, machine vision technology is currently widely used for the online detection of broken and contaminated soybeans during mechanical harvesting. The quality of soybean sample images is a key factor influencing identification accuracy. Existing images typically consist of three channels: the red channel (wavelength range of 600 nm–700 nm) captures long-wavelength light, usually in the red to orange spectrum; the green channel (wavelength range of 500 nm–600 nm) captures medium-wavelength light, covering the green to yellow spectrum; the blue channel (wavelength range of 400 nm–500 nm) captures short-wavelength light, covering the blue to violet spectrum. We found that the RF soybean component classification model, based on the three bands 411.77 nm, 729.25 nm, and 677.54 nm, achieved an overall identification accuracy of 0.9664. In future research, we will use data from these three bands to construct three channel soybean sample images, develop relevant deep learning models, and compare the recognition effects of soybean impurity fragmentation based on different types of wavelength imaging, providing support for the development of efficient and accurate soybean component recognition dedicated image acquisition units.

## 5. Conclusions

A spectral imaging system was used to model and classify soybean components. After preprocessing the spectral data, different RF models based on various preprocessed spectral data were constructed. Through research, we have found that the models based on BC and normalization preprocessed spectra yielded the best results, with the RF soybean component classification achieving an accuracy of 0.9958. In order to reduce the computational complexity of the model, the SPA and CARS methods were used to extract feature wavelengths related to soybean components from the spectral data, and RF models were built based on these feature wavelengths, combined with the best preprocessing methods. For the original spectral data, SPA extracted eight feature bands, achieving an accuracy of 0.9916, while CARS extracted ten feature bands, with an accuracy of 0.9874. On the basis of effectively extracting feature wavelengths, this study further optimizes the recognition model. The RF model was fine-tuned using PSO and DE algorithms, with the DE-optimized RF soybean component classification model achieving the highest prediction accuracy of 1.0000. The RF soybean component classification model built using the top three important bands from DE-CARS had an overall recognition accuracy of 0.9664, while the model built using the top three important bands from DE-SPA-BC achieved a perfect accuracy of 1.0000. The soybean component recognition model studied in this article can effectively classify intact seeds, broken seeds, and impurities, providing technical support for the online detection of soybean quality.

## Figures and Tables

**Table 1:** RF classification results of different spectral preprocessing methods using the prediction set.

Classification Model	Pretreatment	Modeling Band	Complete Grain			Broken Grain			Impurity			Overall Accuracy
			*R*_Precision_	*R*_Recall_	*F*_1_	*R*_Precision_	*R*_Recall_	*F*_1_	*R*_Precision_	*R*_Recal_	*F*_1_	
RF	No	177	0.9867	0.9801	0.9834	0.9375	0.9574	0.9474	1.0000	1.0000	1.0000	0.9790
	BC	173	0.9896	1.0000	0.9948	1.0000	0.9722	0.9859	1.0000	1.0000	1.0000	0.9958
	MA	186	0.9805	0.9934	0.9869	0.9778	0.9362	0.9565	1.0000	1.0000	1.0000	0.9832
	SGD	185	0.9934	0.9934	0.9934	0.9787	0.9787	0.9787	1.0000	1.0000	1.0000	0.9916
	Normalization	162	0.9938	1.0000	0.9969	1.0000	0.9773	0.9885	1.0000	1.0000	1.0000	0.9958
	SNV	184	0.9664	1.0000	0.9829	1.0000	0.8649	0.9275	1.0000	1.0000	1.0000	0.9790
	MSC	189	0.9809	1.0000	0.9904	1.0000	0.9268	0.962	1.0000	1.0000	1.0000	0.9874
	DS	153	0.9756	0.9756	0.9756	0.9677	0.9677	0.9677	1.0000	1.0000	1.0000	0.9916
	SGS	183	0.9805	0.9934	0.9869	0.9783	0.9375	0.9574	1.0000	1.0000	1.0000	0.9832

**Table 2:** RF classification results based on different types of spectral preprocessing and feature wavelength screening.

Classification Model	Feature Wavelength Extraction Method	Pretreatment	Complete Grain			Broken Grain			Impurity			Overall Accuracy
			*R*_Precision_	*R*_Recall_	*F*_1_	*R*_Precision_	*R*_Recall_	*F*_1_	*R*_Precision_	*R*_Recal_	*F*_1_	
RF	SPA	No	0.9934	0.9934	0.9934	0.9787	0.9787	0.9787	1.0000	1.0000	1.0000	0.9916
		BC	1.0000	0.9899	0.9979	0.9714	1.000	0.9855	1.0000	1.0000	1.0000	0.9958
		Normalization	0.9874	0.9812	0.9843	0.9302	0.9524	0.9412	1.0000	1.0000	1.0000	0.9790
	CARS	No	0.9933	0.9868	0.99	0.9583	0.9787	0.9684	1.0000	1.0000	1.0000	0.9874
		BC	0.8713	0.898	0.8844	0.7436	0.6905	0.716	1.0000	1.0000	1.0000	0.9034
		Normalization	0.9758	0.9699	0.9728	0.9038	0.9216	0.9126	1.0000	1.0000	1.0000	0.9622

**Table 3:** RF classification results based on different tuning methods.

Classification Model	Parameter Tuning Method	Feature Wavelength Extraction Method	Pretreatment	Training for Optimal Prediction Accuracy	Validation Set									
					Complete Grain			Broken Grain			Impurity			Overall Accuracy
					*R*_Precision_	*R*_Recall_	*F*_1_	*R*_Precision_	*R*_Recall_	*F*_1_	*R*_Precision_	*R*_Recall_	*F*_1_	
RF	PSO	SPA	BC	1.0000	1.0000	0.9798	0.9898	0.9444	1.0000	0.9714	1.0000	1.0000	1.0000	0.9916
		CARS	No	0.9916	0.9936	0.981	0.9873	0.9464	0.9815	0.9636	1.0000	1.0000	1.0000	0.9832
	DE	SPA	BC	1.0000	1.0000	1.0000	1.0000	1.0000	1.0000	1.0000	1.0000	1.0000	1.0000	1.0000
		CARS	No	0.9916	1.000	0.9873	0.9936	0.9643	1.0000	0.9818	1.0000	1.0000	1.0000	0.9916

**Table 4:** RF classification results based on important feature bands.

Classification Model	Pretreatment	Modeling Band	Complete Grain			Broken Grain			Impurity			Overall Accuracy
			*R*_Precision_	*R*_Recall_	*F*_1_	*R*_Precision_	*R*_Recall_	*F*_1_	*R*_Precision_	*R*_Recal_	*F*_1_	
RF	No	690.42, 925.11, 688.28	0.9688	0.9810	0.9748	0.9455	0.9123	0.9286	1.0000	1.0000	1.0000	0.9664
	BC	411.77, 729.25, 677.54	1.0000	1.0000	1.0000	1.0000	1.0000	1.0000	1.0000	1.0000	1.0000	1.0000

**Table 5:** Identification results of different models.

Classification Model	Pretreatment	Modeling Band	Complete Grain			Broken Grain			Impurity			Overall Accuracy
			*R*_Precision_	*R*_Recall_	*F*_1_	*R*_Precision_	*R*_Recall_	*F*_1_	*R*_Precision_	*R*_Recal_	*F*_1_	
KNN	BC	411.77, 729.25, 677.54	1.0000	0.9873	0.9936	0.9643	1.0000	0.9818	1.0000	1.0000	1.0000	0.9916
SVM	BC	411.77, 729.25, 677.54	1.0000	0.9937	0.9968	0.9818	1.0000	0.9908	1.0000	1.0000	1.0000	0.9958

## Data Availability

Data are contained within the article.
